# Feasibility, Acceptability, and Adoption of Digital Fingerprinting During Contact Investigation for Tuberculosis in Kampala, Uganda: A Parallel-Convergent Mixed-Methods Analysis

**DOI:** 10.2196/11541

**Published:** 2018-11-15

**Authors:** Elizabeth B White, Amanda J Meyer, Joseph M Ggita, Diana Babirye, David Mark, Irene Ayakaka, Jessica E Haberer, Achilles Katamba, Mari Armstrong-Hough, John Lucian Davis

**Affiliations:** 1 Department of Epidemiology of Microbial Diseases Yale School of Public Health New Haven, CT United States; 2 Uganda Tuberculosis Implementation Research Consortium Makerere University Kampala Uganda; 3 Massachusetts General Hospital Center for Global Health Boston, MA United States; 4 Harvard Medical School Boston, MA United States; 5 Clinical Epidemiology Unit, Department of Medicine College of Health Sciences Makerere University Kampala Uganda; 6 Pulmonary, Critical Care, and Sleep Medicine Section Yale School of Medicine New Haven, CT United States

**Keywords:** biometrics, mHealth, mobile phone, tuberculosis

## Abstract

**Background:**

In resource-constrained settings, challenges with unique patient identification may limit continuity of care, monitoring and evaluation, and data integrity. Biometrics offers an appealing but understudied potential solution.

**Objective:**

The objective of this mixed-methods study was to understand the feasibility, acceptability, and adoption of digital fingerprinting for patient identification in a study of household tuberculosis contact investigation in Kampala, Uganda.

**Methods:**

Digital fingerprinting was performed using multispectral fingerprint scanners. We tested associations between demographic, clinical, and temporal characteristics and failure to capture a digital fingerprint. We used generalized estimating equations and a robust covariance estimator to account for clustering. In addition, we evaluated the clustering of outcomes by household and community health workers (CHWs) by calculating intraclass correlation coefficients (ICCs). To understand the determinants of intended and actual use of fingerprinting technology, we conducted 15 in-depth interviews with CHWs and applied a widely used conceptual framework, the Technology Acceptance Model 2 (TAM2).

**Results:**

Digital fingerprints were captured for 75.5% (694/919) of participants, with extensive clustering by household (ICC=.99) arising from software (108/179, 60.3%) and hardware (65/179, 36.3%) failures. Clinical and demographic characteristics were not markedly associated with fingerprint capture. CHWs successfully fingerprinted all contacts in 70.1% (213/304) of households, with modest clustering of outcomes by CHWs (ICC=.18). The proportion of households in which all members were successfully fingerprinted declined over time (ρ=.30, *P*<.001). In interviews, CHWs reported that fingerprinting failures lowered their perceptions of the quality of the technology, threatened their social image as competent health workers, and made the technology more difficult to use.

**Conclusions:**

We found that digital fingerprinting was feasible and acceptable for individual identification, but problems implementing the hardware and software lead to a high failure rate. Although CHWs found fingerprinting to be acceptable in principle, their intention to use the technology was tempered by perceptions that it was inconsistent and of questionable value. TAM2 provided a valuable framework for understanding the motivations behind CHWs’ intentions to use the technology. We emphasize the need for routine process evaluation of biometrics and other digital technologies in resource-constrained settings to assess implementation effectiveness and guide improvement of delivery.

## Introduction

The ability to uniquely identify individuals in health care settings is important for patient care, health system monitoring, and health research. For patients, unique identifiers may facilitate continuity of care, linking of encounters into a longitudinal health record, and prevention of errors during treatment. For health systems, these linkages provide richer evidence for monitoring and evaluation than aggregated data [1]. In clinical and public health research, unique identification helps preserve the integrity of data and protects against misclassification [2]. In resource-constrained settings, however, there are many barriers to unique patient identification: lack of national identification systems, inconsistent spelling of names, uncertainty about date of birth, continually changing phone numbers, a lack of street addresses, and intentional avoidance of identification procedures to escape stigma. A reliable identification method that circumvents these barriers could improve data accuracy and patient retention in care in resource-constrained settings.

Biometric identification techniques offer a novel and appealing solution to these challenges in settings where other identification methods are not feasible or acceptable. Biometric methods rely on an individual’s physical characteristics, such as fingerprints, facial structure, iris geometry, or actions, including handwriting or gait pattern [3]. A number of biometric identifiers, including fingerprint and ocular characteristics, have demonstrated technical feasibility in various studies [4]. However, fingerprint scanning has become the most widely used because of the development and widespread availability of portable, low-cost technologies for digital capture [2] and its high sensitivity and specificity for verification [5]. Others have reported that fingerprinting is feasible [2,5-9] and acceptable [9,10]. However, few published reports exist regarding the actual use of fingerprinting technologies in resource-constrained settings. Therefore, we sought to perform a detailed process evaluation of digital fingerprint scanning by community health workers (CHWs) in urban Uganda to understand the feasibility, acceptability, and adoption of this technology for patient identification [11]. Additionally, we sought to better understand the determinants of CHWs’ intended and actual use of fingerprint scanning technology by applying a widely used conceptual framework, the Technology Acceptance Model 2 (TAM2) [12].

## Methods

### Study Design, Objectives, Setting, and Population

We conducted a parallel-convergent, mixed-methods study of digital fingerprinting in the context of a household-randomized trial of enhanced tuberculosis (TB) contact investigation. Specifically, the trial (called the parent study) sought to evaluate the effects of home sputum collection and short message service text messaging on completion of evaluation for TB among household contacts living with index TB patients. This substudy sought to determine the *feasibility* of digital fingerprinting as measured by the proportion of participants and households successfully identified via fingerprints at baseline and follow-up; to describe the reasons for not capturing fingerprints; and to ascertain the technology’s *acceptability* in principle and *adoption* in practice among CHWs with experience using it.

The parent study was conducted in Kampala, Uganda, from July 2016 to July 2017. In the parent study, we used digital fingerprinting to avoid duplicate registrations of index patients and contacts and to verify follow-up visits at clinics for those needing additional evaluation. Those referred for follow-up evaluation at the clinic included contacts who were persons living with HIV; those who had tuberculosis symptoms but did not produce a sputum sample at the household visit; and those who had an inconclusive diagnostic result for sputum collected during the home visit. All others were not referred for a follow-up visit. In this substudy, we analyzed quantitative data from participants enrolled in the parent study and qualitative data from interviews with CHWs who carried out digital fingerprinting and other study procedures. Children aged <5 years were not eligible for scanning because digital fingerprints are difficult to capture and less accurate in young children [13,14].

### Study Procedures

Prior to implementation, all CHWs completed a course introducing the rationale for the use of fingerprints as biometric identifiers, describing different fingerprint patterns, and training them to capture high-quality fingerprints using a digital scanner. CHWs participated in hands-on training, including “role-play” sessions that allowed them to practice acquiring good-quality fingerprints and troubleshooting commonly encountered problems with fingerprint scanning. All CHWs were trained in infection control practices prior to initiating their work and provided with disposable personal protective equipment to protect them during patient encounters. CHWs performed digital fingerprinting and collected individual age, sex, and self-reported HIV status from household members during contact investigation visits. Fingerprinting was performed using multispectral fingerprint scanners (Lumidigm M301, HID Global, Austin, TX, USA) linked to embedded matching software (Biometrac, Louisville, KY, USA). Matching was available offline and fully integrated as an application programming interface within a customized survey app (CommCare, Dimagi, Boston, MA, USA). The app logged each health worker and time-stamped each encounter. Data were uploaded to a cloud-based server (CommCareHQ, Dimagi). Fingerprint images were not stored but instead recorded as a series of unique characters decipherable only using a secured, proprietary algorithm.

### Quantitative Analysis

For individual contacts, the outcome of interest was the failure to record a complete fingerprint scan in the database, categorized as a binary outcome. A complete scan required successful imaging of the fingerprint with sufficient clarity and resolution to allow adequate feature extraction; scans failing to meet quality criteria (eg, because of degraded ridges, dirt, or fingerpad placement excluding the fingerprint core) were immediately rejected. A complete scan required capture of right and left thumbprints, followed by right and left index fingerprints; any scan that failed to capture all four fingerprints was deemed unsuccessful. Although fingerprinting is an individual procedure, it is frequently offered to multiple household members on a single hardware device during a household visit for contact investigation. To reflect these conditions, we also defined failure at the level of the household encounter; any encounter that did not capture fingerprints from all present household contacts was deemed unsuccessful. If a household required multiple visits to enroll all contacts, we included only the first household encounter in our analyses. Two investigators (EBW and DB) independently reviewed free text explanations from CHWs for fingerprinting failures and classified each as a hardware problem, a software problem, or as another unclassified problem.

We described the population characteristics of individual study participants, including age, sex, and HIV status, as well as characteristics of households, including which CHW captured fingerprints and the time period of enrollment. We examined differences in success by age, using the standard categories employed by the World Health Organization Stop TB Department (5-14 years and ≥15 years); sex; and HIV status. We examined the trend in fingerprinting success over time by the quarter of study enrollment by calculating Spearman rho. In addition, we examined differences in household-level fingerprinting success by CHWs using chi-square test. To test associations between individual characteristics and fingerprinting success, we fit bivariate logistic regression models using generalized estimating equations and a robust covariance estimator to account for clustering by household. We report *P* values based on cluster-robust standard errors (SEs). To estimate the extent of clustering of outcomes by household and CHW, we calculated intraclass correlation coefficients (ICCs).

### Qualitative Interview Procedures

During the last 2 months of the study, we carried out parallel in-depth interviews with each of the 15 CHWs who conducted study procedures using a semistructured interview guide. We developed the interview guide to elicit responses related to 3 overarching topics as follows: the CHWs’ first interactions with digital fingerprinting; their experiences using digital fingerprinting during the study; and their opinions regarding the usability of digital fingerprinting. The guide was developed in English and is reported in Multimedia Appendix 1. One English-speaking investigator (EBW) interviewed all 15 CHWs who conducted study procedures. All but one reported feeling comfortable completing the interview in English; a native Luganda-speaking investigator (JMG) reinterviewed this CHW in Luganda to give the respondent the opportunity to elaborate on experiences and opinions in his or her native language. During the interview, each CHW was also asked to mock-fingerprint the interviewer as a means of eliciting the user’s experiences and interactions with digital fingerprinting. All interviews were recorded, transcribed, and uploaded to a secure Web-based server for qualitative data analysis (Dedoose, Manhattan Beach, CA, USA). In addition, interviewers used a structured debriefing form (Multimedia Appendix 2) to organize emergent themes immediately following each interview. Additional details were added iteratively after reviewing interview recordings and transcripts.

### Qualitative Analysis

**Figure 1:**
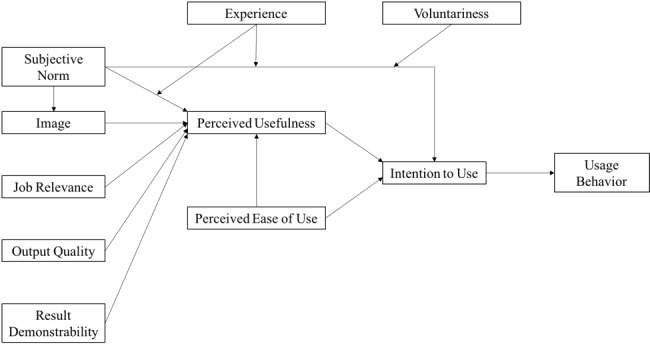
Technology Acceptance Model 2; adapted from Venkatesh and Davis.

We carried out the qualitative analysis using the debriefing forms to identify key themes [15]. Using the TAM2 framework, one investigator (EBW) categorized themes into prespecified antecedents of “behavioral intention” to use fingerprinting technology (Figure 1). TAM2 theorizes that behavioral intention precedes and predicts actual use. Behavioral intentions are influenced by perceptions of the technology’s usefulness and ease of use. Five domains independently contribute to the perceived usefulness of a technology: the perception that important others expect one to use the technology (*subjective norm*); the perception that social status is enhanced through its use (*image*); the perception that the technology supports an important job function (*job relevance*); the performance of the technology (*output quality*); and tangible results of its use (*result demonstrability*) [12,16-19].

### Human Subjects Considerations

Each participant or the parent or guardian of minors provided written informed consent as part of the parent study. Furthermore, participants aged 8-17 years provided written assent. For this substudy, CHWs provided verbal consent prior to the interview. Institutional review boards at the Makerere College of Health Sciences, the Uganda National Council for Science and Technology, and Yale University approved the study protocol.

## Results

### Study Population and Results of Quantitative Analysis

**Figure 2:**
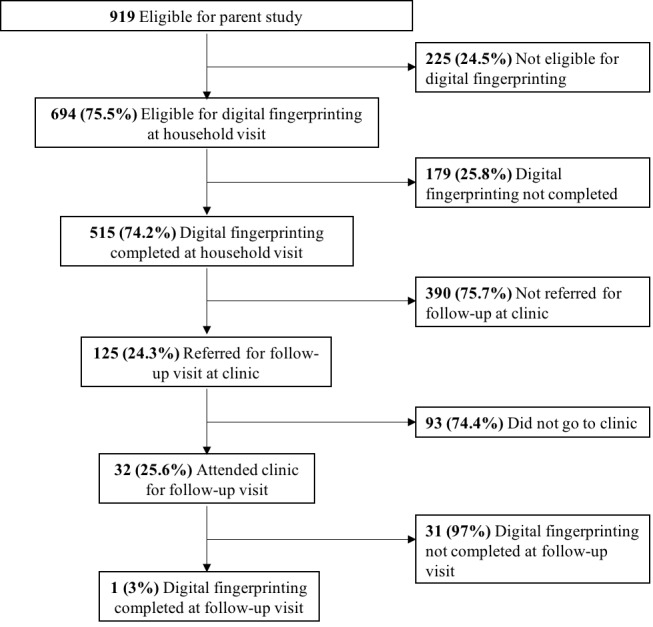
Flow diagram showing enrollment of household contacts.

Of all household contacts eligible for the parent study, 75.5% (694/919) of individuals aged ≥5 years were eligible for digital fingerprinting (Figure 2). Of those eligible, 74.2% (515/694) had a successful fingerprint scan during the household visit. Of the contacts without successful fingerprint scans during the household visit, 60.3% (108/179) of fingerprint scan failures were classified as software problems, 36.3% (65/179) as hardware problems, and 3.4% (6/179) as unclassified problems; none were classified as refusals. We found similar baseline fingerprinting success rates and failure reasons among index patients; because these were individual data collected separately and in a clinic setting, we have reported them separately in Multimedia Appendix 3. Only 3% (1/32) of the contacts fingerprinted at the household visit and referred to the clinic for evaluation were identified via fingerprint at the follow-up visit. Among individual contacts, clustering of unsuccessful scans by household was extensive (ICC=.99). Household contacts who were not successfully fingerprinted did not differ significantly with respect to sex, age, or HIV status from those who were successfully fingerprinted (Table 1).

CHWs successfully fingerprinted all consenting contacts in 70.0% (213/304) of households. Among households, clustering of fingerprint scan outcomes by CHW was modest (ICC=.18). The frequency of successfully fingerprinting all contacts in a household by CHW ranged from 45% to 97%, with a median of 71% (*P*<.001). The proportion of households where all contacts were successfully fingerprinted decreased over time—87% (69/79) in quarter 1, 77% (48/62) in quarter 2, 68% (52/76) in quarter 3, and 51% (44/87) in quarter 4 (ρ=.30, *P*<.001).

### Qualitative Interviews

The CHWs involved in the parent study were recruited on the basis of their high level of previous work experience and their ability to speak both English and Luganda. All 15 CHWs who carried out fingerprint scans were interviewed. The median interview length was 37 minutes (interquartile range: 33.5-42 minutes). CHWs ranged in age from 24 to 54 years with a median of 33 years, and 80% (12/15) of CHWs were females. Most (13/15, 87%) had completed ordinary secondary education (O-Level) or higher and a few (3/15, 20%) had completed university-level education. Most of the CHWs had prior experience using information technology, including smartphones (14/15, 93%), and fewer had previously used computers (8/15, 53%), or tablets (5/15, 33%). All 15 CHWs had worked in a lay health worker role prior to joining the study.

In the interviews, CHWs emphasized how specific experiences with the fingerprinting technology affected their sense of identity, their interactions with household contacts, and their ability to carry out their work. These experiences informed CHWs’ perceptions of the fingerprinting technology’s ease of use and usefulness, two key determinants of intention to use, or acceptance, in the TAM2 model.

**Table 1:** Characteristics of study participants (n=694).

Characteristic		Fingerprint scan (n=515)		No fingerprint scan (n=179)		*P* value^a^	
**Age, n (%)**						.56	
	Children 5-14 years		162 (31.5)		59 (33.0)		
	Adults ≥15 years		353 (68.5)		120 (67.0)		
**Sex, n (%)**						.83	
	Female		336 (65.2)		108 (60.3)		
	Male		179 (34.8)		71 (39.7)		
**Proportion living with HIV, n (%)**						.87	
	Positive		41 (8.0)		17 (9.5)		
	Negative or unknown		474 (92.0)		162 (90.5)		

^a^Corrected for clustering of fingerprint scan outcomes by household with robust SEs.

### Idealized Views of Fingerprinting

CHWs described the usefulness of fingerprinting in an idealized way, reflecting many of the potential applications of fingerprinting that were introduced during training. CHWs consistently said that they believed that fingerprinting would prevent duplicate enrollment and help identify patients who came for follow-up, even if they visited a different study facility.

It’s useful. I get to know exactly I am with the right patient. And if he has ever, for example you have so many facilities, maybe that patient has ever been to [a different health center], and they have ever scanned, so the scanner will refuse or it will tell me already the patient is in the system.

Female, CHW6

Even while acknowledging that the technology did not work perfectly, many CHWs said that they believed that fingerprinting could be useful and should continue.

Me I just wish [the use of fingerprinting] would continue and it could be stable, it could not stop, you know you go to the field and it stops, and you have to do restart, do things, it takes a lot of time.…So me, I just wish in case [fingerprinting] continues, let us do those challenges so we can remove those.

Female, CHW8

By expressing a desire for fingerprinting to continue, despite substantial challenges with the technology, CHWs revealed how much their perceptions of its potential usefulness were driven by their optimism to make it work.

### Positive and Negative Consequences of Digital Fingerprinting for the Self-Image of Community Health Workers

CHWs described their role in the community with pride. They said that they felt that they were providing important services to their patients, whom they often referred to as “clients.” However, fingerprint scanning had complicated implications for CHWs’ self-image. CHWs explained that the technology could both elevate and threaten their social status. On one hand, fingerprint scanning represented an additional service they could offer to their clients, which elevated the capabilities they projected as CHWs. They perceived digital fingerprinting to be an important technology because it is associated with registering for a national identification card and for identification at commercial banks. The excitement of getting to use this important technology in their work helped motivate CHWs to learn and implement fingerprint scanning.

So I was so excited, and I even asked myself, “Who am I, to be in this?” So, I put on my brains in there to really understand what is going to be done. And it took me only two days to get everything in the tablet because I was so attached to it, I wanted it so much.

Female, CHW12

On the other hand, when CHWs struggled to use the fingerprinting technology in front of clients, they felt that their credibility was diminished.

When you’re printing someone and it fails? They just look at you like you don’t know what you’re doing.

Female, CHW13

CHWs placed high importance on their competence in carrying out contact investigation, and a failed fingerprinting attempt could damage one’s credibility. Thus, CHWs perceived that the technology enhanced their social and professional status when it worked smoothly but threatened their status when it failed in the presence of a client.

### Variable Views on the Need and Appropriateness of Digital Fingerprinting

While CHWs generally acknowledged the need for some way to identify patients and contacts to carry out contact investigation, views were mixed regarding whether fingerprinting was necessary. These mixed opinions arose from different perceptions of the job relevance of fingerprinting, or the belief that fingerprinting is important to contact investigation. Some CHWs thought that fingerprinting could be the best way to uniquely identify people:

Even if you give three names, someone might come with, another person might come with three names which are the same. Yet here the fingerprints identify the very person you want.

Female, CHW3

However, others suggested that the name, health center, patient identification number, signatures, photos, or voice recordings would suffice as alternatives. In practice, most CHWs described using some combination of name and other identifiers to identify contacts at follow-up, rather than using the fingerprint. One CHW distinguished between the usefulness of fingerprinting for identifying contacts versus index patients. He said that it was more useful for contacts who are numerous and who come to the clinic months after the CHW meets them. Because index patients are fewer in number, sicker when the CHW meets them, and come back to the clinic often, CHWs felt that they were more memorable and that there was no need to rely on a fingerprint to identify them.

### Impact of Failures to Capture Fingerprints Digitally

Even before interviewers asked about technology failures that prevented the successful capture of fingerprints, CHWs repeatedly turned the discussion toward their experiences with technology failure. CHWs linked the output quality, or how well the technology performed, to their perceptions of its usefulness. A small number of CHWs who reported never having issues with the technology described fingerprinting as being useful. Most CHWs, however, described an increase in technology failures over time, preventing them from capturing fingerprints and adding unnecessary time to the study procedures. When asked whether fingerprinting was useful and should continue in the future, almost all of these CHWs still responded yes, but only if it worked consistently and did not take too much time.

It would be good, like I’ve told you, but the technical issues around it can make the work difficult.

Female, CHW9

Thus, the perceived usefulness of digital fingerprinting depended on it being reliable, fast, and free from technology problems.

### Voluntary Abandonment of Digital Fingerprinting

Most CHWs described instances when they chose to “bypass” the fingerprint scan during contact investigation enrollment; this option was built into the software to allow them to continue with the encounter even when fingerprint scanning failed. They did not indicate any negative impacts of failing to capture a fingerprint on contact investigation procedures. These descriptions suggest that result demonstrability was low and the effect of capturing a fingerprint was not tangible to CHWs.

When it has refused. That’s when I decide to go back and I bypass the fingerprint scanner, and I continue with my patients. I jump it and go to the next question.

Female, CHW5

In addition, CHWs described troubleshooting measures that they used when the fingerprint scanner failed: disconnecting and reconnecting the cable linking the scanner to the tablet, powering the tablet off and back on again, and asking a colleague for help. However, most CHWs said that they only attempted to troubleshoot one to three times—or sometimes not at all—before bypassing the fingerprint scan altogether. In the view of CHWs, whether a fingerprint was successfully captured did not seem to change the contact investigation procedures.

### Variable Confidence in Using the Technology

CHWs differed in their perceptions of the ease of use of the technology, including the scanner itself and the tablet that they used to control the scanner. Some said that it was consistently easy to navigate through the app on the tablet and obtain a fingerprint using the scanner. Others described relying on colleagues or study staff for support when they had problems, which were frequent and which they came to anticipate.

I’m expecting I will go and then I will call [the technology support officer] that this thing has blacked out. So it’s expected…I don’t think I’m the only one complaining about the scanner. They disturb us a lot.

Female, CHW9

This range of comfort with the technology was also reflected during the interview prompt exercise in which CHWs demonstrated the fingerprinting process. Some worked quickly, while others were hesitant when navigating through the app; some were able to describe the process in their own words, while others read directly from the text on the screen. Individuals’ confidence using the tablet and scanner varied greatly.

### Personal Risks to Health Workers

CHWs described two forms of risk that they associated with digital fingerprinting and that influenced their perceptions of its ease of use. First, some CHWs worried about the risk of infection through close contact with patients during the fingerprinting procedure, exacerbated by lack of adequate space and ventilation while performing fingerprinting.

When you’re doing this and this [demonstrating placing fingers on the scanner], you’re kind of getting closer to the patient who is HIV—I mean TB positive, so somehow you are risking. Just try to demonstrate, just try to put your finger here [on the scanner]. So as I’m a community health worker and you have to get closer to me, I’m also breathing in.

Female, CHW9

Second, CHWs said they worried about personal security when carrying the tablet and scanner to household visits.

When we move, some of our places are not in…they are not easy to go there alone. Because you have slums, very dangerous to go with the gadget…And TB is mostly in those places.

Female, CHW7

The risk of infection and lack of personal security introduced psychological and logistical challenges that CHWs had to overcome to carry out fingerprinting.

## Discussion

The inability to uniquely and accurately identify individuals in resource-constrained settings remains a major barrier to improving the quality of health information management and public health research. We found that digital fingerprint scanning was feasible but not reliable—failing to capture fingerprints in about one-quarter of cases—during household contact investigation for TB. Importantly, we found evidence that failures were tightly clustered by household, that they increased substantially over the course of the study, and that there were no systematic differences by clinical or demographic factors. The low rate of fingerprinting at follow-up suggests that CHWs saw little value in the digital fingerprinting system’s usefulness as a verification tool. A systematic qualitative analysis indicated that CHWs continued to find digital fingerprinting acceptable in principle despite the technology’s inconsistent reliability and an accumulating experience with technology failures that decreased their confidence in its usefulness in this setting.

The patterns of fingerprinting failures during the household visit pointed toward problems with the implementation of both software and hardware. Fingerprinting outcomes were almost completely clustered at the household level, suggesting that rather than being driven by sporadic, individual-level failures or refusals, the fingerprinting technology either worked or did not work on a given visit to a household. We identified no individual patient characteristics associated with failure, including age and sex, which argues against degraded individual fingerprints as a cause of failure, as might be expected among adult manual laborers. Furthermore, the predominance of software and hardware problems as explanations for failure and the modest clustering by CHW imply that technology failures were responsible rather than the skills of individual health workers. Finally, the significantly increasing proportion of fingerprinting failures over time reflects the declining functionality of the technology, whether due to health worker disengagement from the technology, software issues, hardware issues or, perhaps, all three.

Previous studies have shown that CHWs without prior experience with digital fingerprinting describe the technology as acceptable in principle [10]. However, we observed that CHWs’ assessments of fingerprint scanning could change as they gain experience with the technology. We found that the TAM2 domains of image, job relevance, and output quality were especially relevant to CHWs’ perceptions of the usefulness of digital fingerprinting in the study. Technology failures lowered CHWs’ perception of the quality of the system, threatened CHWs’ social image, and made the technology more difficult for CHWs to use. Although the technology worked as intended in the majority of interactions, workarounds and a lack of a tangible benefit of fingerprinting ultimately limited its job relevance and perceived usefulness among CHWs. After regular use, CHWs continued to express enthusiasm for fingerprint scanning in principle, but their intention to use the technology was tempered by perceptions that it was inconsistent and of questionable value, ultimately undermining their intention and usage behaviors.

Our findings add to a relatively limited literature on the use of digital fingerprinting for public health applications in sub-Saharan Africa. Our findings differ from a study of the same technology among female sex workers in Zambia, where digital fingerprinting was feasible for and acceptable to clients in the clinic setting, but not acceptable to clients in the field [5]. Perhaps, because participants were at greater risk for stigma or arrest and prosecution, the most common reasons for refusal related to clients’ concerns about a potential loss of confidentiality or privacy. In contrast, we found that a majority of community members underwent fingerprinting during study registration without differences by demographic or clinical characteristics or documented refusals. Similar to a previous study of a mobile health tool for reporting adverse effects of treatments for drug-resistant TB in South Africa, we found that reported enthusiasm for technology—fingerprinting, in this case—did not translate into usage [20]. The acceptance of fingerprinting technology among CHWs serving these clients may decline if they experience technology failures during their work and may be more impactful in terms of its use than the perceived acceptability by community members. There may be a role for communities of practice—learning and peer support networks established to facilitate continuous quality improvement—as patient identification technology is being introduced to help address these challenges [21-23].

Finally, the almost universal failure of lay health workers in this study to use digital fingerprinting at follow-up contrasts with the findings of a study of a biometric identification system for monitoring TB treatment in rural Uganda, which found that fingerprinting improved follow-up among patients engaged in daily directly observed therapy at the clinic [24]. A low background rate of clinic follow-up in our study limited opportunities for digital fingerprinting in this context and, perhaps, therefore, its utility. In settings where digital fingerprinting has been shown to be feasible and acceptable, researchers should conduct larger, well-controlled studies to assess whether fingerprinting is an effective tool for monitoring and improving adherence to follow-up visits in combination with feedback communications. Finally, a limitation of digital fingerprinting is that it is unable to reliably capture fingerprints of children aged <5 years [14], resulting in their exclusion from the analysis. Further studies should evaluate whether newer technologies can accurately capture fingerprints for children aged <5 years. Future studies could also include interviews with household contacts to gain a more comprehensive understanding of the acceptability and challenges of fingerprinting from the perspective of contacts.

This study has a few limitations. First, we had limited data on the technical reasons for each fingerprinting failure. While we were able to categorize failures broadly as related to hardware or software problems, these groupings are not specific enough to guide improvement strategies. Detailed logs itemizing the circumstances of each fingerprinting failure should be included in future evaluations. Second, incomplete data for household- level covariates, such as income, limited our ability to identify predictors of failure to capture fingerprints digitally, although the lack of reported refusals and the very small number of unattributable explanations for failure make patient factors an unlikely explanation.

This study also has several strengths. First, the mixed-methods design enabled complementary analyses of the use of fingerprint scanning during household contact investigation for TB. The quantitative analysis revealed evidence of extensive clustering of failures within household encounters, while the qualitative analysis showed the influence of these failures on CHWs’ perceptions of the technology’s usefulness. Second, we organized key themes offered by CHWs into TAM2 subdomains such as image, job relevance, and output quality, showing how these perceptions shape CHWs’ evolving understanding of the usefulness of fingerprinting technology. Third, we were able to interview the entire CHW population involved in the study rather than relying on a sample. Finally, we evaluated a multispectral fingerprinting technology integrated with and offered as a standard commercial product by a leading global health software platform, increasing the generalizability.

The ability to accurately collect and link individual data to preserve privacy and enhance the generation of quality measures for patients moving through complex care pathways should be a major global health priority [25]. Despite the feasibility and acceptability of biometric identification methods as a means of bringing unique patient identification to resource-constrained settings, the technology we evaluated was not widely adopted by health professionals tasked with using it. As biometric technologies are increasingly introduced in resource-constrained health contexts, our findings point to the importance of theory-informed, mixed-methods evaluation of adoption of these technologies. Mixed-methods data may guide iterative improvements to hardware, software, and the user interface to ensure that the technology aligns with tasks that users find useful and important and engages health workers so that they voluntarily apply the technology to improve the experience of patients. Furthermore, future studies should consider whether detailed process evaluation using mixed methods can be applied to other biometric technologies.

## Appendix

Multimedia Appendix 1 Interview guide used for in-depth interviews with community health workers.

Multimedia Appendix 2 Debriefing form completed by interviewer immediately following each interview, from which emerging themes were identified and analyzed.

Multimedia Appendix 3 Quantitative results for index patients corresponding to those for household contacts that are presented in the Results section.

## Abbreviations

CHW: community health worker
ICC: intraclass correlation coefficient
TAM2: Technology Acceptance Model 2
TB: tuberculosis
