# Supplementary material for: Feasibility, Acceptability, and Adoption of Digital Fingerprinting During Contact Investigation for Tuberculosis in Kampala, Uganda: A Parallel-Convergent Mixed-Methods Analysis
Source: J Med Internet Res. 2018 Nov 15;20(11):e11541. doi: 10.2196/11541 (PMC6265600; doi:10.2196/11541)
Supplement: Multimedia Appendix 1 [file jmir_v20i11e11541_app1.pdf]

Fingerprinting Interview Guide

Introduction to the interview:

- *Introductions between interviewer and CHW*
- *Explanation that the purpose of the interview is to hear about the CHW's experiences using digital fingerprinting in the mHealth study; that there are no right or wrong answers; that if any question is unclear they should feel free to ask for an explanation.*

Consent:

- *CHWs were asked for verbal consent to have the interview recorded.*
- *CHWs were asked for verbal consent for their de-identified responses to be used as part of a research project on digital fingerprinting.*

Introducing question: To begin, can you walk me through the fingerprint scanning process?

First interactions of CHW with fingerprinting technology

Introducing question: Think back to the beginning of the mHealth study. what did you think about the idea of fingerprint scanning when it was first explained to you?

Specifying questions:

- What was it like learning to use the fingerprint scanner?
- If you took time off work, for example, if you took time off for Christmas and then came back to work after a week or two, what was it like starting to use the fingerprint scanner again?

Use of fingerprint scanning during study activities

Introducing question: What is it like using fingerprint scanning with study participants?

Specifying questions:

- What has it been like explaining the fingerprint scanner to study participants? (What has it been like telling the participant about the scanner and why we need to use it?)
    - **Probes:**
        - What is challenging about explaining the fingerprint scanner?
        - What has worked well in explaining the fingerprint scanning process?
        - What do you do when a participant is reluctant to provide a fingerprint?

- What is it like explaining to participants how to place their finger to give a proper fingerprint scan?
    - **Probes:**
        - What part of the fingerprinting process is most challenging?
        - What part of the fingerprinting process works well?
- How do you compare fingerprint scanning at home and at the clinic?
    - **Probe:** Are there differences or is it the same?
- Does the fingerprint scanner ever fail?
    - **Probes:**
        - How does it fail? (What happens when it fails?)
        - What do you do when it fails?

Future use of fingerprint scanning

Introducing question: Moving forward, what do you think about fingerprint scanning in the mHealth study?
Specifying questions:

- What do you think about using fingerprint scanning when you carry out the contact interview?
    - **Probes:**
        - Is it worth using?
        - What alternatives to fingerprint scanning could be used?
- How helpful has FPS been in identifying patients who come back for subsequent visits?
- How would you feel about using fingerprint scanning in general clinic activities?
- Has your opinion of fingerprint scanning changed over time; from the time you first used it to now?

Is there anything else that you find important about fingerprint scanning that we haven't discussed yet?

Do you have any final comments about fingerprint scanning before we finish our discussion?

Have respondent complete the cover sheet

Fingerprinting Study

Community Health Worker Interview Cover Sheet

Interviewer ID: ______________________________ *Respondent ID:* _________________________________

Health Center ID: __________________________ *Initial recruitment contact date:* ___________________

Follow-up #1 (if applicable): __________________*Follow-up #2 (if applicable):* ___________________

Scheduled interview date and time: ____

Scheduled interview location: ____

Respondent information:

Gender: ________________ *Age:* _________________ *Level of education:* ___________________

Length of time involved with mHealth study: ________ ☐ *years* ________ ☐ *months*

Previous experience using technology (check all that apply):

☐ *Computers* ☐ *Tablets* ☐ *Smartphone* ☐ *Other:* ___________________________________

If YES to previous computer experience:

☐ *Personal use* ☐ *Professional use*

Describe: ____

Length of previous computer experience: _______ ☐ *years* _______ ☐ *months*

If YES to previous tablet experience:

☐ *Personal use* ☐ *Professional use*

Describe:

____

_

Length of previous tablet experience: ________ ☐ *years* ________ ☐ *months*

If YES to previous smartphone experience:

☐ *Personal use* ☐ *Professional use*

Describe: ____

Length of previous smartphone experience: ______ □ *years* ____ □ *months*

If YES to other previous technology experience:

□ *Personal use* □ *Professional use*

Describe: ____

Length of other previous technology experience: ___ □ *years* ___ □ *months*

Work position right before mHealth study: ____

____

Recruitment notes

Please use this space to briefly describe how you invited the respondent to be interviewed.

Interview setting notes

Please use this space to briefly describe the setting of the interview.

Field notes

Please use this space and the back of this paper to note any details about the interview that may not be obvious from the recording.
