# Supplementary material for: Feasibility, Acceptability, and Adoption of Digital Fingerprinting During Contact Investigation for Tuberculosis in Kampala, Uganda: A Parallel-Convergent Mixed-Methods Analysis
Source: J Med Internet Res. 2018 Nov 15;20(11):e11541. doi: 10.2196/11541 (PMC6265600; doi:10.2196/11541)
Supplement: Multimedia Appendix 2 [file jmir_v20i11e11541_app2.pdf]

mHealth Fingerprinting Interview Debriefing Tool

Basic Information
**Interview participant:** **Interviewer:** ________<br><br>**Date:**<br><br>**Location:**
**Debriefing**
1. A.) What were your impressions of the discussion?<br><br>B.) Were there topics the CHW seemed uncomfortable discussing or especially passionate about?<br><br>C.) Were there topics the CHW returned to unprompted?<br><br>2. What were the most important themes in the discussion?<br><br>3. First interactions with fingerprint scanning:<br>*A.) Explaining the process*<br><br>*B.) Learning*<br><br>*C.) Retaining*<br><br>4.) Use of fingerprint scanning in mHealth study:

A.) Introducing the scanner to participants

B.) Physically using the scanner

C.) Clinic versus home

5. Future use of fingerprint scanning:

6. Were there any unexpected themes?
