# Supplementary material for: Feasibility, Acceptability, and Adoption of Digital Fingerprinting During Contact Investigation for Tuberculosis in Kampala, Uganda: A Parallel-Convergent Mixed-Methods Analysis
Source: J Med Internet Res. 2018 Nov 15;20(11):e11541. doi: 10.2196/11541 (PMC6265600; doi:10.2196/11541)
Supplement: Multimedia Appendix 3 [file jmir_v20i11e11541_app3.pdf]

Multimedia Appendix 3: Index Patient Results

Figure A1: Flow diagram showing enrollment and digital fingerprinting of index patients

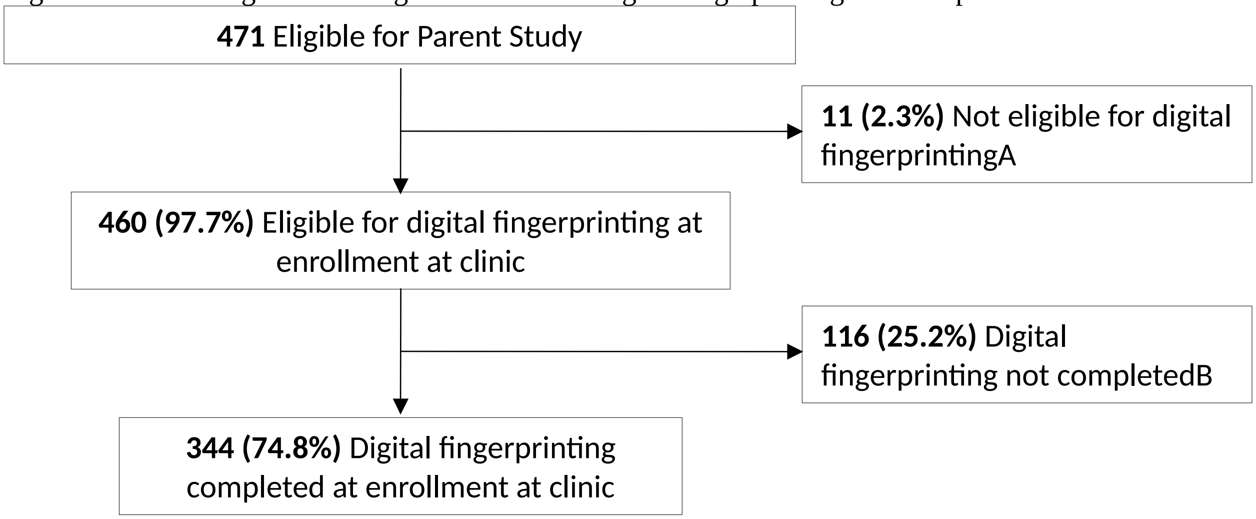

[A] Children under the age of 5 were not eligible for digital fingerprinting.
[B] 64 (55.2%) fingerprint scan failures were classified as software problems, 47 (40.5%) as hardware problems, and 5 (4.3%) as unclassified problems.

Table A1: Characteristics of index patients

**Characteristic n (%)**	**Digital Fingerprint (n=344)**	**No Digital Fingerprint (n=116)**	***P*-value[A,B]**
Age			
Children 5-14 years	10 (2.9)	3 (2.6)	.86
Adults 15 and older	334 (97.1)	113 (97.4)	
Sex (%)			
Female	144 (41.9)	51 (44.0)	.69
Male	200 (58.2)	65 (56.0)	
Proportion living with HIV (%)			
Positive	218 (63.4)	74 (63.8)	.94
Negative or Unknown	126 (36.6)	42 (36.2)	

[A]Chi square test of significance used
[B] *P*-values were not adjusted for clustering by CHW, as clustering was found to be modest (ICC=0.22)
